# Segmental Interaction Energy Controls a Wide Range of Material Behavior

**DOI:** 10.1021/acs.macromol.6c00083

**Published:** 2026-03-26

**Authors:** Ronald P. White, Jane E. G. Lipson

**Affiliations:** Department of Chemistry, 3728 Dartmouth College, Hanover, New Hampshire 03755, United States

## Abstract

In molecular and material modeling, the interaction energy (ε) between segments is a key parameter. Its value is typically specified by connecting experimental thermodynamic behavior, e.g., pressure–volume-temperature (PVT) data, with a model relationship, e.g., a theoretical equation of state (EOS). In this paper, we elevate the practical importance of segmental interaction energies by demonstrating their influence over a surprisingly wide span of behavior, from molecular relaxation and glassification to polymer miscibility. We also quantify an exceptionally strong, direct correlation between ε and the thermal expansion coefficient, which opens opportunities when system data are limited, broadening the scope of thermodynamic characterization to methods such as ellipsometry. Using a variety of experimental data to obtain ε, alongside the EOS prediction for molecular size, we draw bright lines from thermodynamic characterization to predictions for the glass transition temperature, relaxation times associated with the (segmental/structural) α-process, and both activation energies and relaxation times for the small-scale collective motions that enable the newly revealed Slow Arrhenius Process (SAP). The latter is connected to phenomena ranging from stress relaxation and flow to adsorption and crystallization, lipid transfer, and physical aging. We then reverse the path from characterization to prediction by showing that dynamic measurements, specifically on SAP relaxations, can be used to characterize segmental interactions. We demonstrate that the resulting dynamics-derived parameters are effective in making verifiable predictions about polymer miscibility.

## Graphical abstract

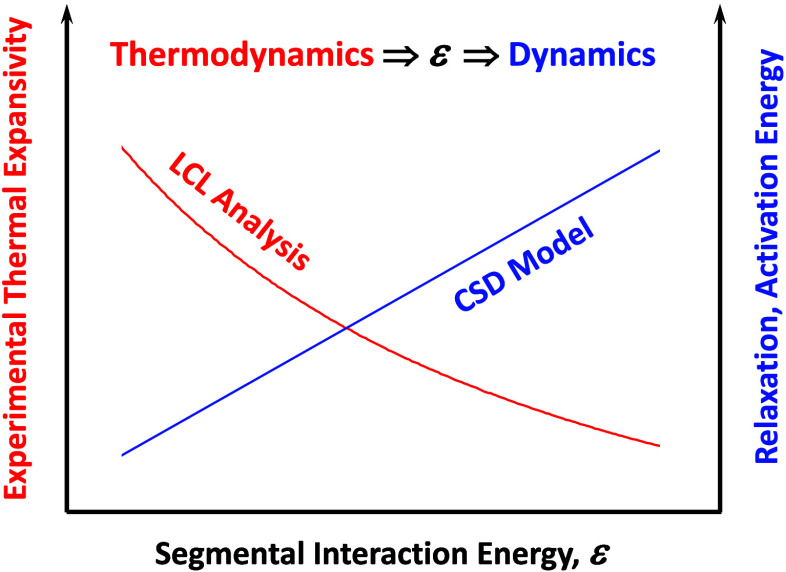

## 1 Introduction

Statistical mechanics-based models that characterize physics occurring at the molecular level can lead to important insights into material behavior and offer the possibility to connect dynamic properties with thermodynamic properties and/or molecular structure.^1–35^ Some of the goals include understanding the temperature and pressure dependence of experimentally observed trends in a material’s molecular level dynamics,^17,18,21,26,29,31,36–42^ the impact of interfaces and additives,^27,43–61^ and also the material response to stresses such as those leading to deformation and flow.^42,62–66^ Even more ambitious are goals such as making predictions about the approach to equilibrium, as in volume or enthalpy relaxation during physical aging,^22,42,67–76^ or when a material crystallizes from the glass,^77,78^ adsorbs on a surface,^79–83^ or mixes/demixes with a completely different material.^84–86^

In this paper we make new progress on several of these wide-ranging goals. We accomplish this through rate models for molecular relaxation^26,31,87^ that rely on thermodynamic characterization of a material via our Locally Correlated Lattice (LCL) equation of state.^25^ In such an approach (more fully discussed below) we have found that one of the most central and insightful parameters is the nonbonded segment–segment interaction energy, ε, which acts between the individual molecular segments. While it arises from thermodynamic modeling, this quantity captures the “energy per movable molecular part” and therefore turns out to make a natural connection with the material’s molecular level dynamics and thus to many kinds of material relaxation rates.

The bridge between ε and predictions about dynamic and thermodynamic behavior is made navigable by what we will show, is the essential connection between this quantity and the coefficient of thermal expansion, α = *V* ^–1^(∂*V*/∂*T*)_*P*_. Although we have a long record of characterizing polymer materials using LCL, Figure 1 is the first time we have plotted α (for 55 polymer melts, i.e., in the liquid state, above *T*_g_) vs the melt’s corresponding molecular segmental energy, ε. (More details are in Section 2 and in the Methods, Section 9.) A very clear trend is revealed, showing how the molecular level property (ε) maps directly to the bulk property (α). The larger a material’s coefficient of thermal expansion, the lower the energy requirement for moving the individual molecular segments of that material, and vice versa. The mapping shown in Figure 1 means that independent experimental determination of α through, for example, dilatometry or ellipsometry or other techniques, will lead directly to a value of ε for the material of interest. As we elaborate below, this provides the key information needed in order to make predictions about such disparate behaviors as miscibility, adsorption, flow, molecular structural relaxation and glass transition, physical aging, and even lipid transfer between membranes.

**Figure 1:**
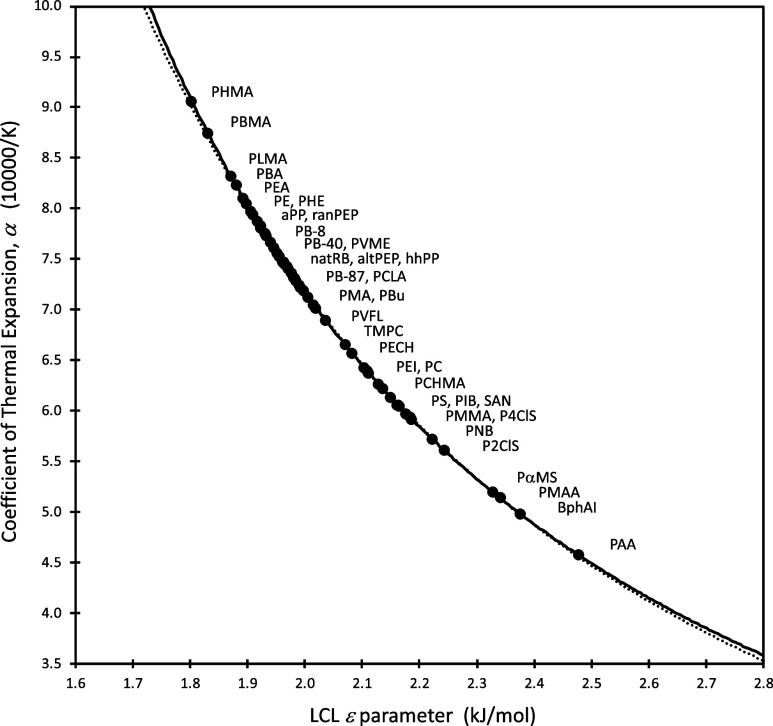
Coefficient of thermal expansion, predicted using the LCL EOS (eq 1) fit to experimental PVT data, plotted against the LCL ε parameter (nonbonded segment-segment interaction energy). A list of polymer names associated with the acronyms can be found in the Methods section. The solid line is the α­(ε) result derived in the text and solved numerically, while the dashed line is the simplified approximation for this relationship described by eq 5.

The paper is outlined as follows: Section 2 delves further into understanding the correlation in Figure 1 and is devoted to recasting the LCL equation of state into a new form that reveals a clear underlying connection between α and ε. In section 3 we explore thermodynamic insights using ε, and elaborate on how some miscibility trends in polymer mixtures can be understood and predicted. In Section 4 we link thermodynamics and dynamics via connections to segmental (α-) relaxation and its most familiar manifestation, the glass transition. In Section 5 we focus on another relaxation route, the recently introduced “Slow Arrhenius Process” (SAP), and demonstrate how thermodynamic characterization through ε leads to predictions for SAP activation energies and relaxation rates. In Section 6, we reveal an approximate pattern that relates a material’s α-relaxation to its SAP. In Section 7 we turn things around to demonstrate how experimental SAP results, say from dielectric or viscosity/rheological measurements, can lead to insight about potential miscibility. Finally, we summarize and conclude in Section 8, followed by method related details made available in Section 9.

## 2 Connecting Bulk Thermodynamic Properties with Molecular Level Parameters

In this section we cover physical insight coming from analysis using the LCL model. The LCL approach falls within the broader general category of statistical mechanics-based equation of state (EOS) models.^1–3,5–10^ In these models the theoretical derivations start at the molecular level (e.g., starting with the partition function) and therefore the model parameters carry a molecular level meaning. The molecular level parameters will typically embody the effects of molecular size and intermolecular interaction energies, such as the LCL ε in regard to the latter. Ideally, once these parameters have been characterized for a given system, the EOS model is then capable of describing a full range of thermodynamic properties. So, knowing the canonical ensemble partition function *Q*(*N*,*V*,*T*), and thus the Helmholtz free energy *A*(*N*,*V*,*T*), standard thermodynamic relations will then lead to the pressure, energy, entropy, chemical potential, etc. We note that liquids (e.g., polymer melts) present a challenge to describe, due to the simultaneous presence of intermolecular attractions alongside the intermolecular repulsions present in the crowded liquid environment. Strategies for dealing with this complexity (i.e., evaluating the amount of available configuration space and adding up the interactions to obtain *Q*), can fall into some basic categories, such as “cell models” and “lattice fluid models”; the Flory, Orwell, Vrij (FOV) model^3^ and the Sanchez and Lacombe (SL) model^6^ are examples of the former and latter, respectively. (See the review in ref 10.) We will be focusing on the LCL model in this paper, as we have found that it yields rich molecular level insight; other models could be similarly interrogated.

The LCL theory describes an experimental system, such as a polymer melt or simpler small molecule liquids and gases, as a fluid of chain-like molecules which are discretized on a lattice. The sites of the lattice can be either occupied by a molecular segment, or vacant. The number of vacant sites can potentially increase or decrease, which thus corresponds to expansion or contraction of the overall fluid system volume. The model’s equation of state expression for the pressure, *P*, as a function of temperature, *T*, volume, *V*, and number of molecules, *N*, is given as follows

1 $$\frac{P}{k_{B}T}=\left(\frac{1}{v}\right)\ln\left(\frac{V}{V-\mathrm{Nrv}}\right)+\left(\frac{3}{v}\right)\ln\left(1-\frac{\mathrm{Nv}}{3V}\left(r-1\right)\right)-\left(\frac{3}{v}\right)\left(\frac{1}{1+\frac{3\left(V-\mathrm{Nrv}\right)}{\mathrm{Nv}\left(2r+1\right)}}\right)\left(\frac{\exp\left[\frac{\varepsilon }{k_{B}T}\right]-1}{\exp\left[\frac{\varepsilon }{k_{B}T}\right]+\frac{3\left(V-\mathrm{Nrv}\right)}{\mathrm{Nv}\left(2r+1\right)}}\right)$$

where *k*_B_ is the Boltzmann constant, and a standard simple cubic lattice coordination value of 6 was taken. In addition to the independent variables, *N*,*V*,*T*, three key molecular parameters are present in eq 1: *r*, the number of effective segments (contiguously occupied lattice sites) per molecule, *v*, the volume per lattice site, and ε, the segment–segment nonbonded interaction energy that was introduced above. Note that together with the interaction energy, ε, the model analysis provides a second fundamentally important molecular level characteristic of a material. This is the volume of a single molecule, which is given by the product, *rv*. Each of the LCL parameters is a constant for a given material, independent of *T, P*, or any other experimental condition. For those interested, the Methods section contains more information on using LCL, including eq 1 written in terms of the more experimentally convenient specific volume, *V*_sp_, and an even simpler approximate expression for *V*_sp_(*T*) at ambient pressure, as well as details for the derivations and development covered here.

The LCL model has a clear definition for the amount of free space that is present in a system. This “free volume”, *V*_free_, is calculated as the difference between the total volume, *V*, and the volume at close-packing, *V*_cp_.

2 $$V_{\mathrm{free}}\left(T,P\right)=V\left(T,P\right)-V_{\mathrm{cp}}=V\left(T,P\right)-\mathrm{Nrv}$$

*V*_cp_ = *Nrv* is the volume of a single molecule (*rv*, the parameter describing the molecular size) multiplied by the total number of molecules (*N*); it is the limiting, lowest possible volume of the model system and thus an important characteristic material constant. A system’s free volume often connects strongly with that material’s behavior. One example is relaxation times (τ_α_) of the α-relaxation process: On isotherms, experimental log τ_α_ values are proportional to 1/*V*_free_, with isotherm slopes increasing as *T* decreases. This qualitative trend is predicted by our Cooperative Free Volume (CFV) rate model for segmental relaxation.^26,31,88^

Related to *V*_free_ is the “fractional free volume”, *V*_free_/*V*, which requires less information to obtain than the absolute value of *V*_free_. At constant ambient pressure (*P* ≈ 0), eq 1 leads to the following implicit relationship for *V*_free_/*V* as a function of temperature.

3 $$\frac{1}{T}=\frac{k_{B}}{\varepsilon }\ln\left[1+\frac{{\frac{3}{4}\left({\left(1-\frac{V_{\mathrm{free}}}{V}\right)}^{-1}-\frac{1}{3}\right)}^{2}}{{\left(-\ln\left[\frac{V_{\mathrm{free}}}{V}\right]+3\ln\left[\frac{2}{3}+\frac{1}{3}\frac{V_{\mathrm{free}}}{V}\right]\right)}^{-1}-\frac{1}{2}\left({\left(1-\frac{V_{\mathrm{free}}}{V}\right)}^{-1}-\frac{1}{3}\right)}\right]$$

Full details on the derivation of this expression are in the Methods section. Notice that the *r* and *v* parameters from eq 1 are not required at all in eq 3; indeed, it is worth highlighting that the only molecular parameter required to predict a material’s fractional free volume (at ambient *P*) is the energetic parameter, ε.

Equation 3 leads to an explanation for the connection, illustrated so clearly in Figure 1, between the coefficient of thermal expansion, α, and the ε parameter. To see this, note that the fractional free volume together with its *T*-dependence at constant ambient pressure (d­(*V*_free_/*V*)/d*T*) relates to α (= *V* ^–1^d*V*/d*T*) via the following

4 $$\frac{d\left(\frac{V_{\mathrm{free}}}{V}\right)}{dT}=\frac{1}{V}\frac{dV_{\mathrm{free}}}{dT}-\left(\frac{V_{\mathrm{free}}}{V^{2}}\right)\frac{dV}{dT}=\alpha \left(1-\frac{V_{\mathrm{free}}}{V}\right)$$

where d*V* = d*V*_free_. According to eq 4, solving for α = (1 – *V*_free_/*V*)^−1^(d­(*V*_free_/*V*)/d*T*) therefore requires only two quantities: *V*_free_/*V*, obtained implicitly from eq 3, and d­(*V*_free_/*V*)/d*T*, obtained from the derivative of eq 3. Note that upon selecting a temperature of interest (e.g., the α values in Figure 1 are for *T* = 425 K), the resulting value for *V*_free_/*V* from eq 3, and also, its derivative, d­(*V*_free_/*V*)/d*T*, will both depend *only* on the value of the system’s ε parameter. This means that ε is the only molecular quantity needed to describe α at any chosen *T*. The relationship illustrated in Figure 1 follows directly from this. Indeed, the solid curve in Figure 1 was generated based on the above, using eqs 3 and 4, where the implicit expressions for *V*_free_/*V* and d­(*V*_free_/*V*)/d*T* (taking *T* = 425 K) were handled numerically.

The α­(ε) relationship of Figure 1 shows a material’s experimentally measured α value can be used to determine ε. In practice it is convenient to apply the following approximate expression

5 $$\alpha \approx {\left[c_{1}\left(\varepsilon^{2}-c_{2}\right)\right]}^{-1}$$

where *c*_1_ = 375.2 K­(kJ/mol)^−2^ and *c*_2_ = 0.2885 (kJ/mol)^2^. The dashed curve in Figure 1 is a plot of eq 5, which agrees well with the full numerical solution. Simply solving eq 5 for the material’s molecular ε parameter based on the experimental α value therefore provides a very convenient and effective route for applying the model and, as subsequent sections describe, making predictions about dynamics. Note the *c*_1_ and *c*_2_ values quoted here correspond to a temperature of 425 K; generally, these values should be sufficient for polymer melt modeling in other *T* regimes as well, as long as this same *c*_1_ and *c*_2_ is applied consistently for all systems; see ref 87 and the Methods section for further discussion and suggestions on implementation.

The above shows that just a single α value is enough to determine the LCL model molecular parameter ε. If one has available data for volume as a function of temperature at ambient pressure, more molecular information is obtainable, including the molecular size, *rv*. We elaborate on this in the Methods section.

## 3 Thermodynamic Trends and Connections with Miscibility

As described in Section 2, alongside the LCL equation of state (eq 1) there are analytic LCL expressions for every other thermodynamic function of interest, and these can all be calculated once a material has been characterized through its values for ε*, r*, and *v*. This can be readily accomplished by applying eq 1 to experimental PVT data, a route that yields robust characterization that can be used to derive insight and make predictions about both pure component and mixture behavior. In Figure 2 we introduce another new connection (alongside that of Figure 1) between bulk thermodynamics and LCL’s molecular level characterization, showing the material’s internal cohesive energy (*E*) divided by its absolute entropy (*S*) plotted against ε for a large set of polymers (*T* = 425 K). The detailed expressions for the LCL internal energy, entropy, and Helmholtz free energy, are all available in the Appendix of ref 25. (Note that in LCL, the “internal energy” (in magnitude) is the same as the “cohesive energy”, i.e., it is a purely attractive energy. In the lattice formalism, repulsions are effectively infinite and so do not contribute to the internal energy.)

**Figure 2:**
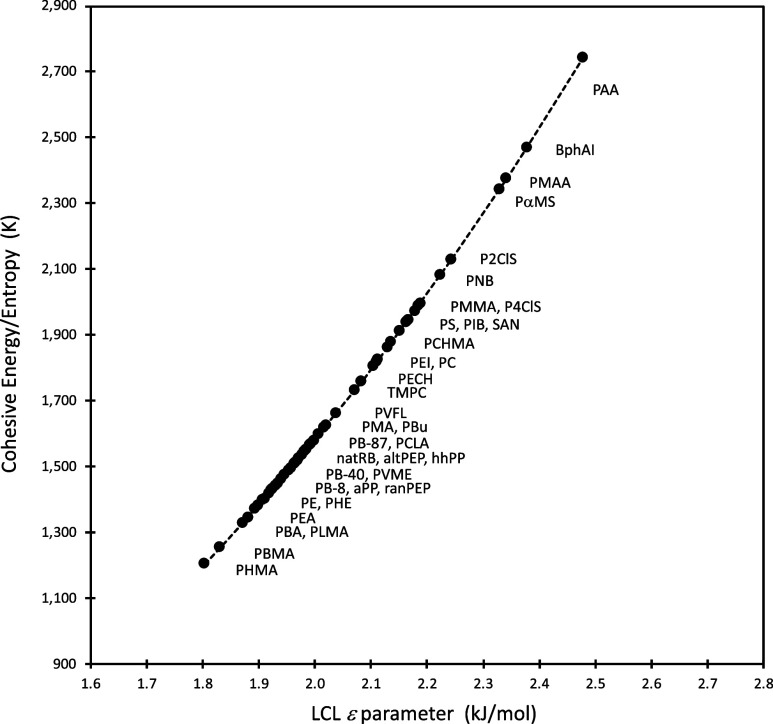
Ratio of LCL calculated cohesive energy to entropy (at *T* = 425 K and constant ambient pressure) plotted against the LCL ε parameter. A list of polymer names associated with the acronyms can be found in the Methods section. The dashed line is a guide to the eye.

A key takeaway from Figure 2 is that the ε parameter dictates that material’s energy per degree of freedom or, as stated in the Introduction, ε is the material’s energy per molecular moving part, and this is why (see examples further below) ε has such a strong connection to the material’s dynamics. ε contributes to, but does not directly correlate with the bulk cohesive energy of the material; for example, some materials have a strong cohesive energy (per mass or volume) but just a modest value for ε; in such scenarios, the entropy is larger, and LCL has determined (per mass or volume) that the material has a larger number of freely moving segments (more degrees of freedom). To illustrate this point, it is instructive to contrast the ε parameter more explicitly with the bulk cohesive energy and bulk cohesive energy per volume, i.e the “cohesive energy density”.

The cohesive energy density (CED) is defined as CED = *E*/*V*. This well-known metric can be expressed directly in terms of the molecular parameters when we consider the CED at the simplifying limiting condition of close packing. At close packing, we can write the energy as *E*_cp_ = (1/2)­(*z* – 2)*Nr*ε = 2*Nr*ε, showing that *E*_cp_ is proportional to both ε and the total number of segments, *Nr*. Here we took the polymeric (large *r*) limit, the *z* – 2 factor subtracts out the two bonded connections on each segment, where *z* = 6 is the simple cubic lattice coordination number, and the usual factor of 1/2 corrects for double counting of segmental interactions. Taking *V*_cp_ = *Nrv* from above, we obtain a simple expression for the cohesive energy density at close packing: *E*_cp_/*V*_cp_ = 2ε/*v*. We see that while the energy per volume (the CED) relates to ε/*v*, the energy per entropy (Figure 2) relates to just ε alone.

The energy per degree of freedom (ε) is also linked to effects on mixture miscibility, through its correlation with α (Figure 1). A strong mismatch between component α values can lead to unfavorable compressibility effects on mixing. The link with ε suggests that the two components are experiencing significantly different entropy gains upon expansion, relative to the cost in lost cohesive energy.

We apply these results to reveal some notable trends within families. For example, replacing the ester methyl group in PMA with an ester ethyl group, to give PEA, weakens the segmental interaction, ε, by about 125 J/mol, as well as *E*/S by about 250 K. However, those numbers grow 3-fold when undertaking the same replacement in the *backbone* alkyl substituent, from methyl in PMMA to ethyl in PEMA, leading to reductions of about 375 J/mol and 750 K in those two quantities. The impact of changing from a methyl to an ethyl is substantially greater when the alkyl group is on the backbone, as compared to when it completes the ester linkage on the side chain. In both cases increasing the alkyl chain length likely creates more flexibility, both interfering with local, and weakening, segmental interactions.

Other insights that derive from this figure can lead to explanations for miscibility. For example, PIB is a well-known outlier in terms of miscibility among polyolefins. All of the other widely used polyolefins have *E*/S values that cluster between 1400 and 1500 K which rationalizes the tendency for them to mix as illustrating the principle of “like dissolving like”. PIB is very different; the significant short branching per chemical repeat leads to an *E*/S value of over 1900K. Another way to think of this difference is through the related ε values, which are clustered around 2000 J/mol for the rest of the cohort, but closer to 2200 J/mol for PIB. Combining this insight with that from Figure 1 shows that PIB has a markedly smaller thermal expansion coefficient than the other polyolefins. As noted above, a significant mismatch in thermal expansion coefficient between two materials is characteristic of partial miscibility which, in fact, is associated with a Lower Critical Solution (LCST) temperature. A large enough ε mismatch will likely cause total immiscibility, because there will be negative volume changes, and thus unfavorable entropy changes, upon mixing. We can actually go further, and identify how |ε_11_ *–* ε_22_| differences can be of LCST versus UCST (Upper Critical Solution Temperature) miscibility. We will come back to this in Section 6.

As a final remark on miscibility we note the distinction between our treatment of molecular level interaction parameters (ε) compared to the well-known Flory–Huggins χ parameter. Specifically, and unlike ε, the χ parameter reflects no pure component properties and is used as a mixture fitting function, typically both temperature and composition dependent.

## 4 Relating Thermodynamic Analysis to the α-Relaxation and the Glass Transition

We now show how the ε parameter, obtained from thermodynamic data, relates to the dynamics of segmental relaxation through the so-called α-process. In the α-mechanism a segment shifts a distance on the order of its own diameter, into a space cooperatively created by neighbors, as part of a characteristic relaxation mechanism. This contributes to the path by which a glassy solid forms a glassy melt, i.e., it is a path to structural relaxation and maintaining equilibrium upon changes in *T*, *P*, etc. While segmental relaxation occurs over a range of time scales and temperatures, we begin by connecting to one characteristic relaxation temperature, the glass transition, *T*_g_. For all that follows note that we view the glass transition (*T*_g_) as marking a *dynamic* event, i.e., it is not interpreted here as any kind of a “thermodynamic transition”.

### 4.1 How the Segmental Interaction Energy (ε) Relates to *T*_g_

In a recent paper, Wolf et al.^89^ investigated the glass transition temperatures in a series of small molecule liquids, correlating them with corresponding physical properties. In that work they applied ellipsometry to determine the coefficient of thermal expansion of each species. Note that film height (*h*) is measured in these ellipsometry experiments, not specific volume, so the approach characterizes the system only through its α value. When a polymer film sample is supported on a substrate, and is in its melt state above *T*_g_, it expands like a fluid in a piston and so its film height will change with temperature such that *h* ^–1^d*h*/d*T* is equal to α = *V* ^–1^d*V*/d*T*.^90,91^ In this scenario where only α is available we can still apply the simple eq 5 (α – ε relationship) to determine each system’s ε value from the experimental α value. These new results for small molecule ε values, based on ellipsometry experiments, are shown in Figure 3, and compared with corresponding trends in ε for polymeric systems.

**Figure 3:**
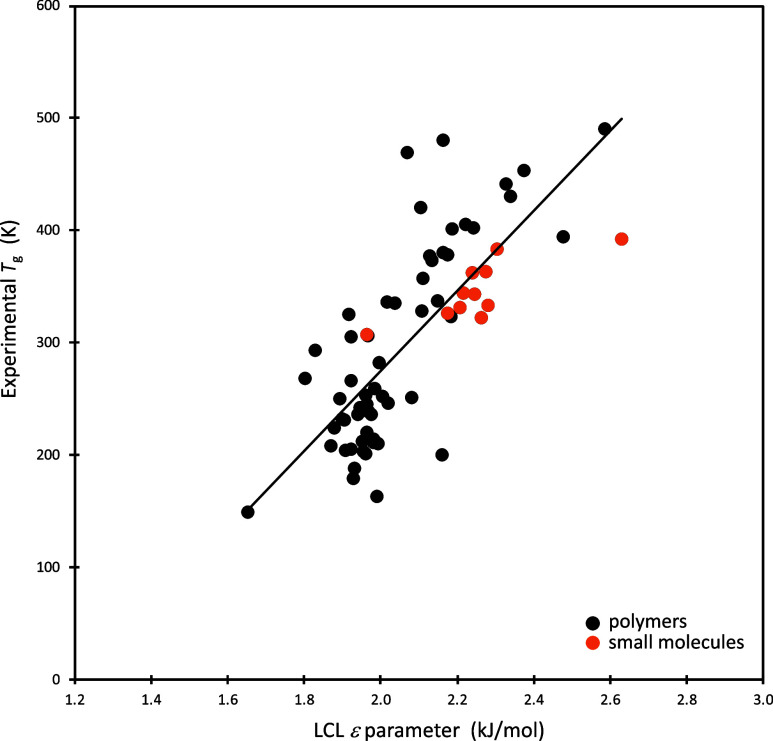
Experimental glass transition temperatures (*T*_g_) plotted against the LCL ε parameter for that material. Black symbols are for polymers, orange symbols are for small molecules. The line is a “best fit”.

The small molecule results appear in the figure as the orange symbols, and exhibit a notable correlation between ε and the corresponding system *T*_g_ value. The black symbols in the figure represent results using our analysis of thermodynamic data on a large set of polymer melts (see Methods section). The combined set shows that for both small molecules and polymer melts there is a general trend whereby the increasing strength of nonbonded segment–segment interactions, as reflected in the ε parameter, corresponds to a slow down in the α-relaxation process and a resulting increase in the glass transition temperature. Connecting this observation with the discussion of Figure 2 leads to predictions for glass transition trends within certain families, for example increasing the *n*-alkyl chain length in a polyacrylate, whether as part of the ester linkage or on the repeat unit backbone (in a poly *n*-alkyl acrylate) might be expected to reduce the glass transition temperature of the polymer; experimental evidence shows that this is indeed the case.^92^

We note that the small molecule data suggest what might be a separate line of lower slope, although that perception is dominated by the two outerlying points. There is obviously considerable spread, overall, so while it makes sense to leave open the possibility that small molecules may indeed define a different slope, additional data are needed. Minus those two outerlying points, the majority of small molecule results fit comfortably into the larger trend. We have seen some examples where tighter correlations were found within a more restricted family of systems; in those cases, the correlations were between *T*_g_ and fractional free volume, the latter of which (as shown in Section 2) is closely connected to ε. For example, tighter trends were found within the family of polymethacrylates,^93^ and within the polyacrylates,^92^ and within a series of cross-linked poly vinyl ethylenes;^94^ the latter series does have a characteristic slope that differs considerably from the average over all systems.

### 4.2 α-Relaxation Times and the Thermodynamics-based Cooperative Free Volume Model

One experimental route to study the slow down in structural dynamics (α-relaxation) as a glass-forming system approaches its *T*_g_ is via dielectric spectroscopy (DS),^95,96^ a method that can provide information about segmental relaxation that spans decades of time, over a broad range of temperatures and pressures. A common, dynamic, definition of *T*_g_ in this context is the temperature, at ambient pressure, associated with a relaxation time, τ_α_, of about 100 s. We have seen above that thermodynamic characterization can be linked to *T*_g_ through ε. Deeper connections can be made by exploiting our full LCL characterization which together with ε will also yield the close-packed molecular size (*rv*) and ultimately, the free volume, *V*_free_, as defined by eq 2. It is worth emphasizing that *V*_free_ is a LCL *prediction* (eq 2) because *r* and *v* are both characteristic material parameters that are part of our fundamentally derived equation of state.

Approaching segmental relaxation as a kinetic event that can be addressed in terms of transition state theory we have derived the Cooperative Free Volume (CFV) model and demonstrated that, although it is a thermodynamic quantity, *V*_free_ is a natural variable for analyzing and predicting segmental relaxation as a function of both temperature and pressure. This turns out to work extremely well for a broad range of glass forming materials, both in the bulk^26,31,88^ and in thin films.^50,97^ The CFV equation is given by

6 $$\ln\tau_{\alpha }=\left(\frac{V_{\mathrm{cp}}}{V_{\mathrm{free}}}\right){\left(\frac{T^{*}}{T}\right)}^{b}+\ln\tau_{\mathrm{ref}}$$

where *b*,T*,τ_ref_, are material specific parameters; here we use the relative free volume, *V*_free_(*T*,*P*)/*V*_cp_. According to the CFV model prediction, verified for a wide variety of polymeric and small molecule materials,^26,31,88^ relaxation data (log τ_α_) plotted as isotherms should yield straight lines, with slopes that increase with decreasing *T*. The strength of this *T*-dependence will depend on the scaling parameter *b*.

In Figure 4 we present new results, illustrating how well the CFV model holds for poly­(methyl acrylate), PMA. These data span a temperature and pressure range of 300 to 360 K and 0 to over 300 MPa, respectively. The main panel shows that the CFV prediction of linear log τ_α_ vs 1/*V*_free_ isotherms with *T*-dependent slopes is very well obeyed. The upper left inset also demonstrates how the entire set of results collapse onto a single line using *b*.

**Figure 4:**
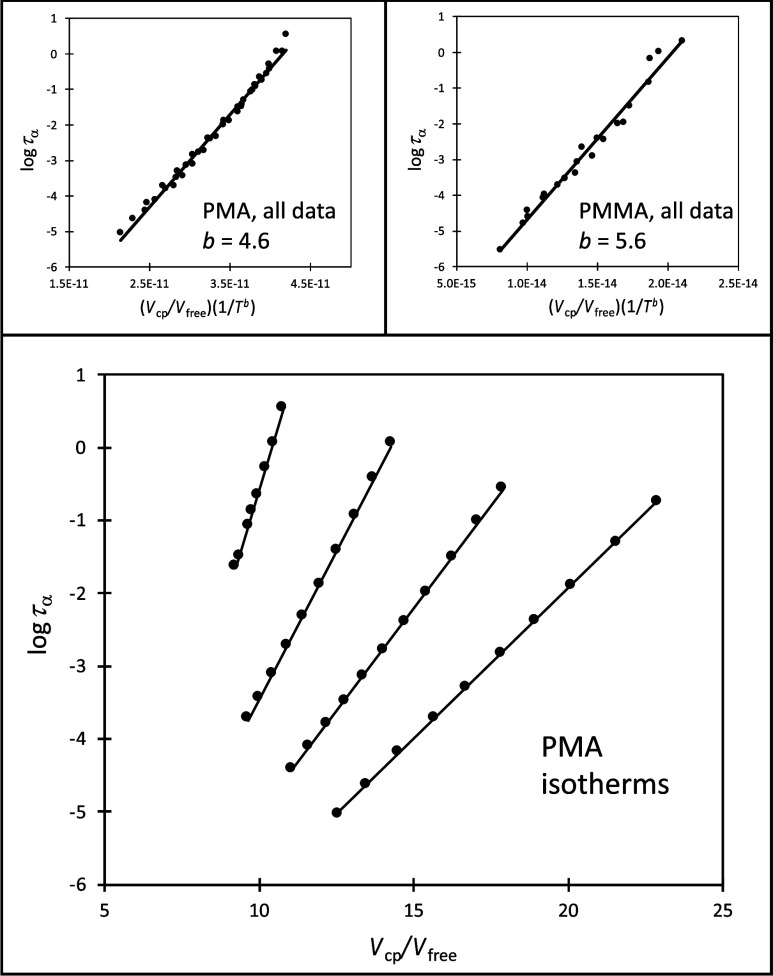
Lower panel shows experimental dielectric spectroscopy data for *T*,*P*-dependent α-relaxation times (logτ_α_) for PMA (poly­(methyl acrylate)) plotted as isotherms against the LCL prediction for inverse relative free volume. Temperatures from top to bottom are 301, 321, 340, 360 K. Upper two panels are for PMA (left) and PMMA (poly­(methyl methacrylate), right) showing the data collapse when all relaxation times are scaled by (1/*T* ^*b*^) where “*b*“ is the CFV parameter determined through application of eq 6 to the experimental data for each polymer. For all three panels the lines represent best fits. The experimental dynamics data are taken from ref 98 (PMA) and ref 99 (PMMA). The LCL predictions for the corresponding *T*,*P*-dependent *V*_free_ value at the *T*,*P* of each dynamics datum point was based on model characterization to experimental PVT data taken from ref 100.

In fact, in addition to PMA, the upper right inset shows this collapse for a second newly analyzed system, poly­(methyl methacrylate), PMMA. The two polymers have repeat unit structures that differ only by a methyl group attached to the backbone, which leads us to explore how such a change might be reflected in dynamic response. From eq 6 we find

7 $$b={\left(\partial \;\ln V_{\mathrm{free}}/\partial \;\ln T\right)}_{\tau }$$

In words, this means that *b* characterizes how *V*_free_ and *T* must change in concert, in order to keep a material’s relaxation time fixed, in the same way that *P* and *T* must change in a coordinated fashion in order to keep the volume of a gas constant. A smaller *b* signifies a material has greater sensitivity to free volume changes, since a smaller shift in free volume is needed to counter a degree’s increase in temperature so as to maintain a fixed relaxation time.

We had previously determined *b* values spanning about 1.5 to 8.4 for a wide range of polymer and small molecule glassy systems^31,94^ and found PPMA to be about 6.3. Now analyzing both PMMA and PMA for the first time, we find *b* = 5.6 for the former and 4.6 for the latter. Going from PMMA to PPMA, i.e., changing the ester methyl to a propyl, yields a material that needs a larger change in free volume, per degree change in temperature, in order to maintain a fixed relaxation time (i.e., it is less sensitive to free volume change than to temperature change). However, keeping the ester structure fixed but removing the backbone methyl, in going from PMMA to PMA, shifts *b* to a lower value, with a smaller change in free volume needed to counter the effect of temperature increase so as to keep the relaxation time fixed (so PMA has increased relative sensitivity to free volume compared to PMMA). Using segmental relaxation time as a diagnostic, the first example of structural change reduces the material sensitivity to free volume changes, whereas the second example increases it.

Other theoretical models for *T*,*P* dependent dynamics characterize sensitivity of segmental relaxation to volume (not free volume) changes, for example, via the γ parameter in the density scaling formalism.^29,36,39–41^ The values reported^98,101^ for γ indicate more volume sensitivity for PMA (it has a higher γ = 2.55) than for PMMA (γ = 1.8), and this is analogous to our conclusions above. In fact, CFV can *predict* system γ values, and we test this on the two newly analyzed systems here. Applying the relationship derived in ref 31, which uses the *b* parameter value, and the value of the fractional free volume at *T*_g_, we predict γ = 2.47 and 1.64 for PMA and PMMA respectively, which is in excellent agreement with the above literature values that were based on density scaling.

## 5 Relating Thermodynamic Characterization to the Slow Arrhenius Process

In this section we extend our discussion involving the thermodynamic segmental energy, ε, to other kinds of dynamics properties and processes. In particular, we consider the so-called slow Arrhenius process (SAP),^81,102–104^ a type of molecular level motion that has recently become reliably measurable via dielectric spectroscopy (DS) techniques.^95,96^ The SAP has been shown to be important in driving a number of material equilibration processes such as adsorption and crystallization.^77,82^ Furthermore, the onset regime of physical aging also appears to be driven by the SAP rather than the α-process (at *T*’s ≤ *T*_g_ – 5K); here experimental evidence shows that the activation energies for these aging onset times match the activation energy of the SAP determined via DS.^81^ The SAP also appears to be closely connected to polymeric shear flow at high temperature,^81,105^ and it has been appearing in other rheological experiments at lower temperatures.^106–109^ We have formulated a model for the rate of the SAP,^87^ and from that one can model the rate of any experimentally observable process (shear flow, adsorption, crystallization, physical aging) that may be driven by it; the rates of these observables have been found to be proportional to the DS measured rate.^77,81,82^ In fact, we have also successfully used this new model to analyze data on, and make predictions about, lipid transfer between vesicles and lipid membranes.^110^ As indicated above, this “collective small displacements” (CSD) model creates another link between the thermodynamic segmental energy, ε, and dynamic processes; the CSD is described below.

### 5.1 The Segmental Energy, Viscous Flow, and the Slow Arrhenius Process

We consider first the connection of the thermodynamic segmental energy, ε, with the activation energy for flow, *E*_flow_, at high temperature, about 150 degrees above *T*_g_. Such *E*_flow_ values come from the temperature dependence of various rheological observables, such as from direct measurements of shear viscosity, shift factors from oscillatory experiments, etc. Shown in Figure 5a, for a dozen polymer melts, is the correlation between *E*_flow_ and the segmental energy, ε, obtained based on each melt’s coefficient of thermal expansion, α (via eq 5). It should be emphasized that the two quantities plotted in the figure are based on completely different kinds of experiments, one is a measurement of dynamics, and the other is based on thermodynamics. The result is a strong connection: as the strength of the segmental energy increases, so does the activation energy for flow. This analysis therefore provides a path for modeling and understanding rheological and related properties based on thermodynamic properties.

**Figure 5:**
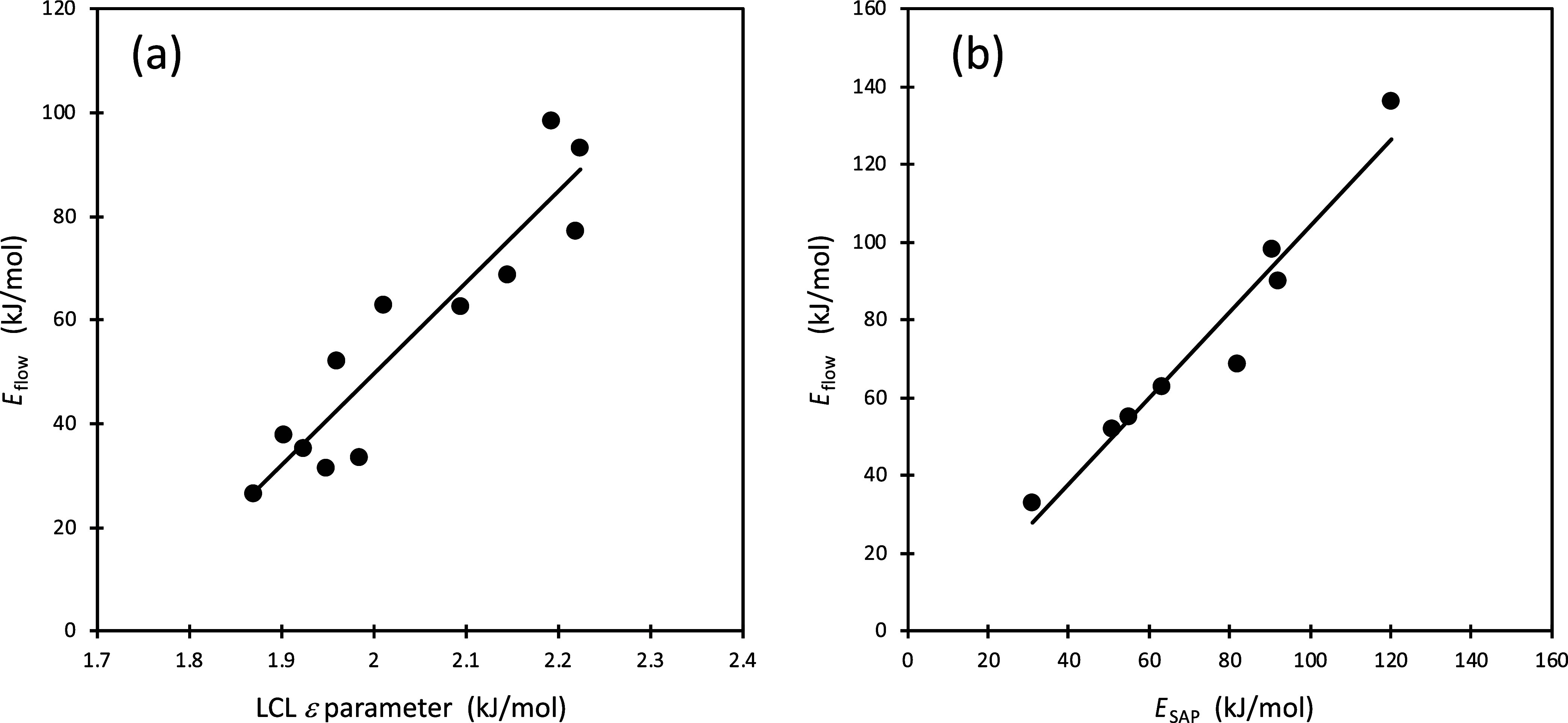
Activation energy for flow at high temperatures (*E*_flow_) plotted in panel (a) against the LCL ε parameter obtained via EOS (eq 1) fit to experimental thermodynamic data for each polymer. See ref 87 for a list of the polymer systems. In panel (b) the plot of *E*_flow_ is against the experimentally determined SAP activation energies from dielectric relaxation spectroscopy. See Methods section for list of polymers. Experimental data for *E*_flow_ are from refs 66,111–113 , and for *E*_SAP_ from refs 81,103 In both panels the lines represent best fits. The plot in panel (a) is adapted from Figure 3 of ref 87.

We now turn to the slow Arrhenius process (SAP) and start by highlighting one of its important connections to rheological measurements. Specifically, Figure 5b is a plot of the experimentally determined activation energy for flow at high *T* (*E*_flow_) for a number of polymer melts against the corresponding activation energy of the SAP (*E*_SAP_), determined via dielectric spectroscopy. The tight correlation with slope of unity shows that the two activation energies, *E*_SAP_ and *E*_flow_, are, in fact, the same. This relationship, and the correlation between *E*_flow_ and thermodynamics (Figure 5a) are both key in the CSD model for the SAP, which is described next.

### 5.2 Collective Small Displacements Model for the Slow Arrhenius Process

The Collective Small Displacements (CSD) model was inspired by several important observations known about the SAP. One is the fact that the SAP can function at temperatures both above and below *T*_g_. In the latter regime large segmental hops (which would require significant cage breaking) such as those in the α-process cannot be occurring to any significant degree on the laboratory time scale. So, in CSD, the motion of the SAP is pictured as the collective movement of a group of effective segments such that all individual movements are small, less than a segmental diameter. The segments in the group move in such a way that they collectively deform their own local amorphous packing, but without any large hops; this local segmental packing deformation may be shear-like, consistent with the observed connection to shear flow. Though the segmental movements are modest, the change induced in the local amorphous packing is enough to relax local stress, realign local structures, and locally consolidate free space allowing volume relaxation. This can therefore drive changes in material observables, as those seen connected to the SAP in rheology measurements (stress relaxation), physical aging (volume consolidation/relaxation), adsorption and crystallization (realignment) and even lipid transfer.

The CSD model expression was derived based on a simple transition state theory picture. Here the group of collectively moving segments must become activated (softened) by thermal fluctuations, and both the energy change and entropy change to obtain this activated state are important.^87^ The resulting expression for the relaxation time, τ_CSD_ (= τ_SAP_ via DS), of the small-scale collective molecular motions as a function of temperature, *T*, (at ambient pressure) is given by the following

8 $$\tau_{\mathrm{CSD}}=\tau_{\mathrm{oo}}\exp\left[-\left(\frac{m\left(\varepsilon -\varepsilon_{o}\right)}{k_{B}T_{\mathrm{oo}}}\right)\right]\exp\left[\frac{m\left(\varepsilon -\varepsilon_{o}\right)}{k_{B}T}\right]$$

where *m*(ε – ε_o_) is the activation energy, *E*_CSD_ (= *E*_SAP_ = *E*_flow_)­

9 $$E_{\mathrm{CSD}}=m\left(\varepsilon -\varepsilon_{o}\right)$$

A number of examples (Arrhenius plots) showing eq 8 predictions for experimentally measured relaxation times (τ_SAP_) as a function of *T* are available in ref 87. Note that in eqs 8 and 9 the parameters, *m* = 177 (±23),^87^ ε_o_ = 1.72 kJ/mol, *T*_oo_ = 435 K, and τ_oo_ = 0.0238 s, are all taken to be material independent, and were obtained in the original work^87^ based on the analysis of a large set of materials. (While the τ_oo_ = 0.0238 s prefactor value applies to DS, to apply the CSD model to equilibration kinetics, just a single experiment will yield the analogous prefactor in the equation equivalent to eq 8 for that chosen experimental technique; all the other parameters are invariant.) This leaves a single material dependent parameter, the segmental energy, ε, determined from thermodynamic analysis. It is important to emphasize that because all CSD parameters other than ε are reasonably material independent, relaxation times for the SAP (τ_SAP_) can be predicted based solely on thermodynamic information. Specifically, all that is needed to use model eqs 8 and 9 is a coefficient of thermal expansion (α), which leads to the ε parameter via eq 5. No dynamics data are required at all.

A demonstration of this thermodynamics-based approach is Figure 6, which shows a plot of thermodynamic α values vs activation energies determined via experiment (points) together with the activation energies predicted by CSD (curve) using eq 9. The CSD predictions (*E*_CSD_) are in excellent agreement with both the activation energies of the SAP measured via DS (*E*_SAP_), and with rheological activation energies (*E*_flow_) for over two dozen polymers. This is further highlighted in the inset, which gives the ratio of *E*_SAP_/*E*_CSD_ and *E*_flow_/*E*_CSD_; the ratio is near unity for all systems.

**Figure 6:**
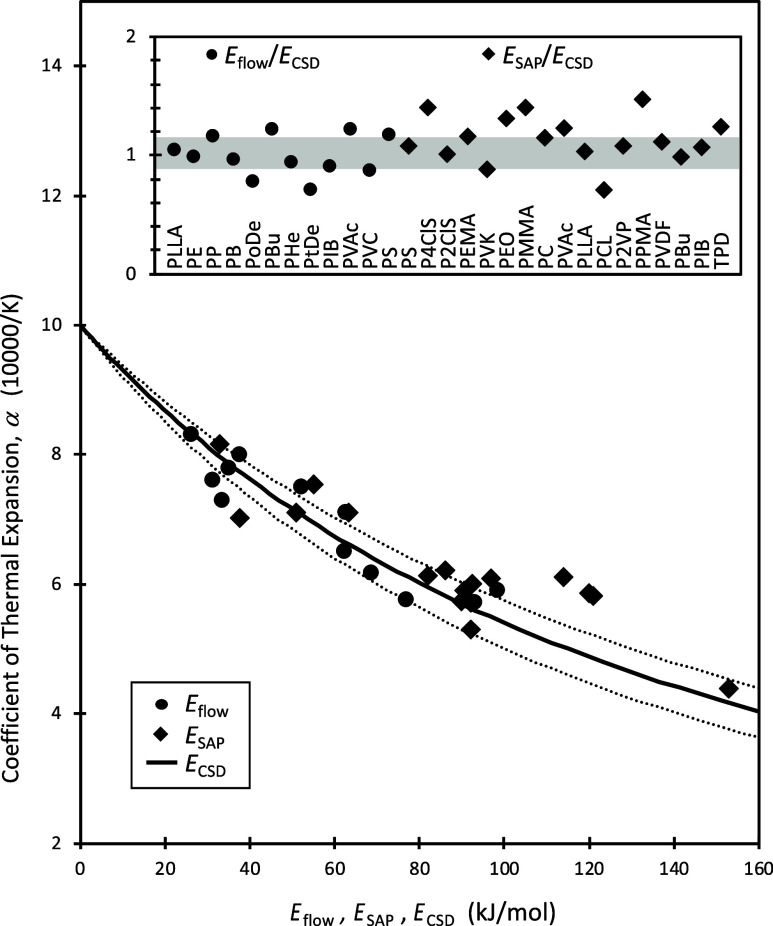
Main panel: The coefficient of thermal expansion plotted against the activation energies from flow experiments (*E*_flow_, circles), dielectric spectroscopy experiments on the SAP (*E*_SAP_, diamonds), and the solid line (*E*_CSD_) is the CSD model prediction, while the dashed lines give the boundaries proscribed by the error bar for the CSD parameter *m* = 177 ± 23. The inset illustrates that *E*_CSD_ is in consistently strong agreement with the different experimental activation energies, given that the ratio of experimental to theoretical values is close to one for the entire set of polymers studied. The shaded bar reflects the error in *m*. Experimental data for *E*_flow_ is from refs 66,111,112 , and for *E*_SAP_ from refs 81,103,104 . See Methods section for more system info. Note our extrapolation of the CSD model to intersect the ordinate is not intended as a prediction for what to expect at zero activation energy.

The ability to map between the segmental interaction energy, ε, and the coefficient of thermal expansion, α, has also been recently exploited to predict dynamics in a number of other applications. In simulation results the ε parameter was used for a series of model polymeric systems of varying α to make CSD-based predictions of each system’s *E*_flow_.^83^ In the crystallization of glassy small molecule systems, the ε parameter was obtained from α values determined via ellipsometry, leading to CSD-based predictions for activation energies and crystal growth rates,^77^ and characterization of ε has also led to prediction of polymer adsorption rates.^87^ Finally, the effective ε of lipid vesicle membranes was obtained based on *T*-dependent density measurements leading to the prediction of rates of lipid transfer in vescicles.^110^

## 6 Predicting the Connection between the SAP and *T*_g_

We have seen in Section 4 that the ε parameter connects with the α-relaxation (*T*_g_), and, in Section 5, that the ε parameter connects with the slow Arrhenius Process (SAP). This therefore suggests that there is some link between the α-relaxation and the SAP. Here we formulate an approximate expression relating *T*_g_ to the activation energy of the SAP.

The CSD model predicts *E*_SAP_ = *m*(ε – ε_o_), or, ε = ε_o_ + *E*_SAP_/*m*. The correlation line in Figure 3 shows that ε = *k*_1_(*T*_g_ + *k*_2_), where *k*_1_ = 2.81 × 10^–3^ kJ/mol/K, and *k*_2_ = 437.4 K. Equating these two relationships involving ε leads to the following new relationship

10 $$T_{g}\approx \frac{E_{\mathrm{SAP}}}{mk_{1}}+\frac{\varepsilon_{o}}{k_{1}}-k_{2}$$

A plot of *T*_g_ vs *E*_SAP_ is shown in Figure 7. The line shown is the prediction of eq 10, which tracks fairly well through the symbols representing the actual experimental results for a number of systems. The scatter about the prediction line is mostly attributable to the scatter about the average relationship between *T*_g_ and ε. A few data points^114^ for an SAP-like mechanical response found in metallic glasses (square symbols) have also been included in the plot.

**Figure 7:**
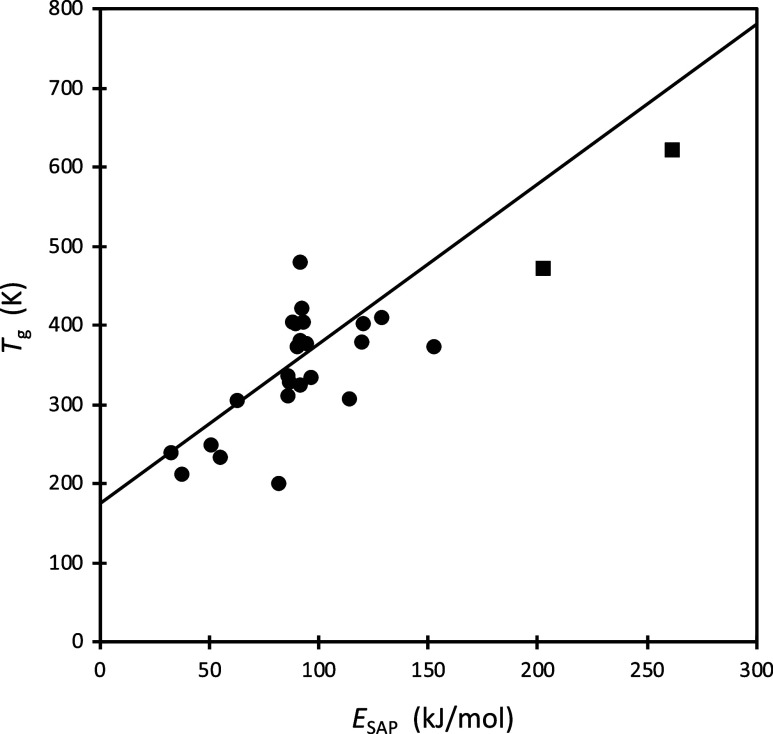
Experimental *T*g values plotted against experimental *E*_SAP_, illustrating the relationship proposed in eq 10 (solid line). Circles represent polymers and three small molecules; a list of these systems is provided in the Methods section. Squares represent metallic glasses^114^ exhibiting an SAP-like mechanical response.

Note that the results of eq 10 and Figure 7 imply not only an approximate connection between *T*_g_ and the SAP, but also between *T*_g_ and viscous polymer flow. Other forms of correlations between *T*_g_ (α-relaxation) and polymer flow (terminal relaxation) have appeared in the past, such as the correlations of van Krevelin,^115^ however, direct connections have been elusive given that experimental tests of *T*-dependence show some differences between α-relaxation times and overall chain/terminal relaxation times.^18^ Our evidence here does point to some potential for a common thermodynamic origin.

## 7 Predicting Mixture Thermodynamics Based on Dynamics Measurements

Finally, before summarizing and drawing some larger conclusions, we consider using dynamics results, in this case for the SAP, as a route to providing new insight about thermodynamic behavior. This reverses the path we have taken in previous sections. Given experimentally determined SAP values for *E*_*SAP*_ we can use eq 9 in Section 5.2 (wherein *E*_SAP_ = *E*_CSD_ = *m*(ε – ε_o_)), along with the quoted values for the constants *m*, and ε_o_ to determine ε for a polymer of interest. While this yields only one of the three LCL characteristic parameters, it is enough to predict the material’s coefficient of thermal expansion, and fractional free volume. Moreover, prior work has shown that considering the difference in pure component segmental interaction energies, |ε_11_ – ε_22_|, can lead to significant insight about potential miscibility behavior.

There are two typical categories involving partial miscibility in polymer mixtures: blends exhibiting a UCST tend to be in the lower molecular weight range, with values in the tens of thousands, or thousands, of g/mol. For these mixtures the enthalpy of mixing is positive (opposing miscibility), while the ideal entropy of mixing, which is also positive and favors miscibility, typically plays a significant role. Therefore, excess entropy of mixing contributions, Δ*S*_xs_, can be important in shifting the free energy balance. We have previously demonstrated^116,117^ that in the LCL theory this quantity is controlled by |ε_11_ – ε_22_|; for small energetic differences the LCL predictions for Δ*S*_xs_ can be positive or negative, but we have found that they become increasingly unfavorable (negative) as the difference between pure component ε values grows.

In the case of LCST blends the enthalpy of mixing is negative, while the overall entropy of mixing is also negative, which means Δ*S*_xs_ is exclusively negative. This aligns with our conclusions from studying dozens of blends, which is that large disparities in pure component ε values, i.e., large |ε_11_ – ε_22_|, are correlated with partial miscibility of the LCST type; typical LCST ε differences are in a range around 150 J/mol.^116^ Note that this is J, not kJ. If the ε difference is sufficiently large, e.g., 300 J/mol or more,^116^ it will probably lead to complete blend immiscibility. Conversely, small values of |ε_11_ – ε_22_| suggest partial miscibility of the UCST type, and our prior work^116^ suggests values in the range of 50 J/mol or less are likely to fall into this category. However, if the ε values are very close, leading to |ε_11_ – ε_22_| that is very small, then even if a slightly negative Δ*S*_xs_ is predicted, it is unlikely to dominate the favorable enthalpy and ideal entropy contributions, and so such a blend would probably be fully miscible.

In this section we will apply our prior experience in using the SAP results in order to partially characterize, and then consider the possible blending, of polystyrene (PS) and polyisobutylene (PIB). We have not previously studied this combination as a potential blend. Published measurements on these polymers have yielded SAP activation energies of *E*_SAP_ (PS) = 90.4 kJ/mol and *E*_SAP_ (PIB*)* = 82.0 kJ/mol. Applying eq 9 with *m* = 177 and ε_o_ = 1.7 kJ/mol yields ε_PS_ = 2.21 kJ/mol and ε_PIB_ = 2.16 kJ/mol, giving |ε_PS_ – ε_PIB_| = 50 J/mol, a value which clearly points in the direction of UCST behavior. It turns out that there are reports of PS/PIB phase separation in the literature: A 1992 study by Wu et al.^118^ of an oligomeric mixture of PS and PIB under flow reports UCST phase separation, while a slightly later study^119^ of a somewhat less oligomeric mixture reported a UCST (cloud point) of about 335 K. This sample case shows that dynamic SAP characterization of pure materials can be used in conjunction with thermodynamic models to yield insight regarding equilibrium thermodynamic behavior.

Given this is the first such application of SAP data it makes sense to ask how the material ε values derived this way compare with values from LCL analysis of PVT data. And, even if the actual ε values are not a strong match, how |ε_PS_ – ε_PIB_| compares, since there could well be systematic discrepancies in individual ε values obtained in such extremely different ways. LCL analysis of PVT data (Table 1) yields ε_PS_= 2.14 kJ/mol and ε_PIB_ = 2.16 kJ/mol, with their difference being ∼ 25 J/mol. For this example we see very good agreement between LCL and SAP results (both predict small |ε_11_ – ε_22_|), suggesting that SAP data may be a promising route for a partial thermodynamic characterization of materials under study.

**Table 1:** Polymer Characterization Results - Molecular Parameters and Acronyms^a^

acronym	full name	*r*/*M*_w_ (mol/kg)	*v* (mL/mol)	ε (J/mol)	⟨*T*⟩ of fit range (K)	PVT data refs
PS	polystyrene	0.1153	7.562	2136	423	^100^
P4ClS	poly(4-chloro styrene)	0.0968	7.647	2187	433	^100^
P2ClS	poly(2-chloro styrene)	0.0924	7.930	2244	433	^123^
PαMS	poly(α-methylstyrene)	0.1088	7.764	2329	478	^124^
PIB	polyisobutylene	0.1139	8.985	2162	430	^100^
PE	polyethylene	0.1428	7.521	1907	424	^100^
altPEP	poly(ethylene-*co*-propylene) alternating	0.1248	8.640	1964	429	^100^
ranPEP	poly(ethylene-*co*-propylene) random	0.1284	8.377	1924	430	^100^
aPP	atactic polypropylene	0.1185	9.064	1924	429	^100^
hhPP	head-to-head polypropylene	0.1175	8.958	1966	429	^125^
aPBu	atactic poly(1-butene)	0.1064	9.942	2020	432	^100^
iPBu	isotactic poly(1-butene)	0.1070	10.060	2007	430	^100^
POc	poly(1-octene)	0.1121	9.354	1910	431	^100^
cisPB	cis polybutadiene	0.1238	8.310	1991	428	^100^
PB-8	polybutadiene (8% 1–2 addition)	0.1355	7.541	1931	430	^126^
PB-24	polybutadiene (24% 1–2 addition)	0.1316	7.784	1933	430	^126^
PB-40	polybutadiene (40% 1–2 addition)	0.1231	8.317	1957	430	^126^
PB-50	polybutadiene (50% 1–2 addition)	0.1201	8.502	1953	430	^126^
PB-87	polybutadiene (87% 1–2 addition)	0.1052	9.842	1986	430	^126^
PI-8	polyisoprene (8% 3–4 addition)	0.1123	9.162	1993	430	^126^
PI-14	polyisoprene (14% 3–4 addition)	0.1218	8.367	1981	430	^126^
PI-41	polyisoprene (541% 3–4 addition)	0.1214	8.389	1977	430	^126^
PI-56	polyisoprene (56% 3–4 addition)	0.1254	8.139	1963	430	^126^
natRBR	natural rubber	0.1304	7.746	1963	429	^100^
PAA	poly(acrylic acid)	0.1243	5.206	2478	424	^100^
PMA	poly(methyl acrylate)	0.1189	6.372	1999	428	^100^
PEA	poly(ethyl acrylate)	0.1121	7.255	1894	426	^100^
PPA	poly(n-propyl acrylate)	0.1092	7.845	1940	425	^100^
PBA	poly(*n*-butyl acrylate)	0.1150	7.585	1881	426	^100^
PMAA	poly(methacrylic acid)	0.1297	5.435	2341	446	^100^
PMMA	poly(methyl methacrylate)	0.1107	6.958	2178	424	^100^
PEMA	poly(ethyl methacrylate)	0.1095	7.410	2017	381	^100^
PPMA	poly(n-propyl methacrylate)	0.1028	8.255	1969	381	^100^
PBMA	poly(*n*-butyl methacrylate)	0.1440	5.898	1831	427	^100^
PHMA	poly(*n*-hexyl methacrylate)	0.1420	6.266	1803	423	^100^
PCHMA	poly(cyclohexyl methacrylate)	0.1049	7.873	2129	424	^127^
PLMA	poly(lauryl methacrylate)	0.1346	7.174	1872	424	^100^
PDMS	poly(dimethylsiloxane)	0.0846	10.931	1655	429	^100^
PEO	poly(ethylene oxide)	0.1494	5.416	1900	423	^100^
PECH	polyepichlorohydrin	0.0905	7.480	2083	403	^10^
PC	polycarbonate	0.1181	6.372	2105	433	^100^
TMPC	tetramethyl bisphenolA polycarbonate	0.1292	6.180	2072	495	^128^
PPO	poly(phenylene oxide)	0.1034	7.964	2166	503	^100^
PES	poly(ether sulfone)	0.0992	6.713	2589	522	^100^
PEI	poly(ethylene isophthalate)	0.1340	5.086	2109	424	^100^
BphAI	bisphenol A isophthalate	0.1031	7.286	2377	478	^100^
PNB	polynorbornene	0.1133	7.960	2224	435	^100^
PVFL	poly(vinyl formal)	0.1317	5.689	2038	426	^100^
PVBL	poly(vinyl butyral)	0.1329	6.251	1918	426	^100^
PVF	poly(vinyl fluoride)	0.1054	6.689	2151	488	^100^
PVDF	poly(vinylidene fluoride)	0.0934	5.908	1972	458	^100^
PVC	poly(vinyl chloride)	0.1089	6.023	2113	409	^100^
PVME	poly(vinyl methyl ether)	0.1115	7.930	1946	424	^129^
PVAc	poly(vinyl acetate)	0.1229	6.279	1923	427	^100^
PCLA	polycaprolactone	0.1270	6.659	1984	424	^100^
PLLA	poly(l-lactide)	0.1116	6.562	2184	411	^100^
SAN	poly(styrene-*co*-acrylonitrile)	0.1229	6.921	2164	423	^100^

a Parameters and values are defined in the text. The *r* parameter is tabulated here as *r*/*M*_w_ where *M*_w_ is the polymer molecular weight. Note the characterization results shown here are for fitting to PVT data sets (e.g., not to just α via eq 5), and the data used in these fits came from particular temperature ranges, with an average *T* given by the ⟨*T*⟩ value listed. Other characterizations and comparisons, e.g., focusing around a different ⟨*T*⟩, can give somewhat different values. Therefore, as emphasized in the main text, for consistency in any given model comparison it is best to try to keep ⟨*T*⟩ similar through all systems studied if possible. Note finally that when an experimental *M*_w_ value is not available, we fit the PVT data assuming a polymeric value, such as *M*_w_ = 100,000 g/mol; fitted *r*/*M*_w_ values will all be similar for any choice of *M*_w_ as long as it is within the polymeric regime, e.g., *M*_w_ > 10,000 g/mol.

## 8 Summary and Conclusions

In this paper we show that the interaction energy between effective molecular segments, ε, plays a leading role in understanding and predicting material behavior ranging from dynamic relaxation, to adsorption and crystallization, to polymer miscibility. ε is a fundamental characterization parameter, typically obtained via analysis of thermodynamic data, e.g., using our model LCL equation of state. On the thermodynamic side, we introduce and explain revealing connections between ε and the system’s thermal expansion coefficient as well as its cohesive energy per unit entropy. Exploiting the former allows us to introduce a strong correlation between ε and the glass transition temperature. Shifting to dynamic behavior, we use our model (CSD) for the slow Arrhenius process (SAP) and connect ε to activation energies and relaxation times for phenomena that can range from viscous flow to crystallization, adsorption, aging, and even lipid transfer. Further, we show linkage between the SAP and the more familiar segmental α-relaxation process; our model for the latter (CFV) has demonstrated predictive power using the related LCL characterization of the close packed molecular volume (*rv*) to obtain *V*_free_. Finally, we discover that reliable ε values can be extracted from SAP dynamic relaxation results. For the first time this allows us to bypass the usual requirement for thermodynamic data. Focusing on a blending pair we have never studied, we use SAP-derived ε values and predict likely UCST phase separation, which we verify using results from the experimental literature. Overall, we wish to close by emphasizing the close interdependence between thermodynamic and dynamic properties, and how this helps in understanding the behavior of materials ranging from small molecule to polymeric.

## 9 Methods

In this section we derive some thermodynamic relations that follow from the *P*(*N*,*V*,*T*) equation of state expression in eq 1. This includes a simple relationship between temperature and volume at ambient pressure, which is shown to depend on just two effective parameters, the interaction energy, ε, and the molecular size, *rv*. Ultimately, this simplification leads to eq 3, and then to the connection between ε and α in Figure 1. Further below, for the convenience of those interested in applying the LCL model to actual experimental data, we provide the equation of state relationships (eqs 1 and 11) written in terms of specific volume (rather than absolute volume), and, we provide some practical suggestions on model implementation. Finally, we provide a Table of LCL characterizations and info for a large set of polymers.

To start, we consider the condition of constant ambient pressure (*P* = 1 atm), and note that in the context of a liquid, atmospheric pressure is essentially zero pressure. In particular, starting with the *P*(*N*,*V*,*T*) expression in eq 1, we set *P* = 0, and also take the polymeric limit of large *r* where *r* – 1 ≈ *r* and 2*r* + 1 ≈ 2*r*. This leads to the following simplification.

11 $$\frac{1}{T}=\frac{k_{B}}{\varepsilon }\ln\left[1+\frac{\frac{3}{4}{\left(\frac{V}{\mathrm{Nrv}}-\frac{1}{3}\right)}^{2}}{{\left(\ln\left[\frac{V}{V-\mathrm{Nrv}}\right]+3\ln\left[1-\frac{\mathrm{Nrv}}{3V}\right]\right)}^{-1}-\frac{1}{2}\left(\frac{V}{\mathrm{Nrv}}-\frac{1}{3}\right)}\right]$$

This equation is a good approximation when the goal is just to describe the ambient pressure *V*(*T*) behavior of a liquid or polymer melt. Here *T*(*V*) is expressed analytically making eq 11 an implicit expression for *V*(*T*). In practice, *V*(*T*) can be readily solved numerically using *T*(*V*). To appreciate the utility of eq 11, note that in practical applications, it might commonly be the case that only ambient pressure *V*(*T*) data are available for a material of interest. However, by fitting that limited ambient data to eq 11, one can still obtain the material’s fundamental segmental interaction energy (ε parameter), and, the material’s effective molecular size (the product *rv*).

Note that in eq 11 the *r* and *v* parameters only appear together as the product *rv*, allowing it to be treated as a single effective parameter. The product *rv* is all that is needed to predict/calculate free volume, *V*_free_ (see eq 2). Taking this one step further, there is also the option of replacing the appearance of *rv* in eq 11 altogether, by writing in terms of fractional free volume, *V*_free_/*V*. Specifically, when we substitute (*V* – *Nrv*)/*V* = *V*_free_/*V* and *Nrv*/*V* = 1 – *V*_free_/*V* into eq 11, the result is the simple *T*(*V*_free_/*V*) relationship discussed above (eq 3), wherein just the single molecular parameter, ε, remains, leading to the direct connection between ε and α that was demonstrated in Figure 1.

As noted above, for convenience we provide the full equation of state expression (eq 1) written in terms of specific volume, *V*_sp_, as follows

12 $$\frac{P}{\mathrm{RT}}=\left(\frac{1}{v}\right)\ln\left(\frac{V_{\mathrm{sp}}M_{w}}{V_{\mathrm{sp}}M_{w}-\mathrm{rv}}\right)+\left(\frac{3}{v}\right)\ln\left(1-\frac{v}{3V_{\mathrm{sp}}M_{w}}\left(r-1\right)\right)-\left(\frac{3}{v}\right)\left(\frac{1}{1+\frac{3\left(V_{\mathrm{sp}}M_{w}-\mathrm{rv}\right)}{v\left(2r+1\right)}}\right)\left(\frac{\exp\left[\frac{\varepsilon }{\mathrm{RT}}\right]-1}{\exp\left[\frac{\varepsilon }{\mathrm{RT}}\right]+\frac{3\left(V_{\mathrm{sp}}M_{w}-\mathrm{rv}\right)}{v\left(2r+1\right)}}\right)$$

and the corresponding ambient (zero) pressure volume-temperature relationship (eq 11) written as

13 $$\frac{1}{T}=\frac{R}{\varepsilon }\ln\left[1+\frac{{\frac{3}{4}\left(\frac{V_{\mathrm{sp}}M_{w}}{\mathrm{rv}}-\frac{1}{3}\right)}^{2}}{{\left(\ln\left[\frac{V_{\mathrm{sp}}M_{w}}{V_{\mathrm{sp}}M_{w}-\mathrm{rv}}\right]+3\ln\left[1-\frac{\mathrm{rv}}{3V_{\mathrm{sp}}M_{w}}\right]\right)}^{-1}-\frac{1}{2}\left(\frac{V_{\mathrm{sp}}M_{w}}{\mathrm{rv}}-\frac{1}{3}\right)}\right]$$

The inputs and suggested units for these equations are as follows. *M*_w_ is the molecular weight in g/mol, and *R* is the gas constant in J/K/mol, where, for *T* in K, *V*_sp_ in mL/g, and *P* in MPa, ε should be in J/mol, and *v* and *rv* should be in mL/mol (i.e., *v* is the segmental volume per mole of segments, and *rv* is the molecular volume per mole of molecules), where *r* is unitless. Following from eq 2, free volume, per gram of sample, is equal to *V*_sp_ – *rv*/*M*_w_, where *rv*/*M*_w_ is the close-packed volume per gram of sample. Analogous to what was noted about eq 11, ambient pressure experimental *V*_sp_(*T*) data can be fit to eq 13 to obtain the two effective parameters, ε and *rv*/*M*_w_. For those interested in using LCL, note that there was a typo in eq 13 of ref 25. The eq 1 expression for the pressure here in this work, and its *V*_sp_ based variant (eq 12), are the correct expressions. Also note that earlier publications describing LCL model expressions often follow a convention where the ε parameter is defined as a negative quantity, while here it is positive, i.e., it is the magnitude of the attractive interaction.

As LCL is a model for fluids, model characterization is most often applied to experimental data on the material’s melt/liquid state. When implementing the model, as we have emphasized before,^31,87,116,120^ it is important to keep in mind that the fitted molecular parameters will depend somewhat on the temperature range applied. This is traceable to the fact that simple theoretical equations of state commonly have a *T*-dependence of the coefficient of thermal expansion that is too strong. For consistency, we suggest that all of the systems to be compared in any model investigation are fit to the same temperature range of experimental data. That is, try to choose a data range that has about the same average *T* for all systems even if the data range is small. Obviously there can be limitations in how close a match can be achieved, e.g., due to restrictions of data availability, and how some systems may have high glass transition or melting temperatures which limits the melt ranges.

Note these cautions on model fitting apply not only for LCL, but for other EOS’s too. All simple EOS’s exhibit a fitting range dependence in their parameters. Some tests of LCL parameter dependence on fitting range were shown in ref 116. Relatedly, there are several papers^25,121,122^ where plots are available showing LCL model curves plotted together with the corresponding fitted PVT data, which gives a sense for how well the curves hold at the edges of the fitting ranges; the quality of the LCL fits are comparable to (if not better than) those that one would obtain with, for example, the FOV and SL models. In light of the inevitable deviations, it should likewise be kept in mind, that the advantage of using simple EOS’s is that their molecular level parameters will better maintain a clear physical meaning (because these parameters are limited in number, typically just three).

Following the above suggestions about fitting to consistent data ranges will make the model molecular parameters and predicted properties the most comparable from one system to the next. A good recent example of this is when LCL was applied to inform the CSD dynamics model.^87^ A wide range of systems needed to be compared, so, we obtained all ε values (which had led to CSD predictions for the SAP activation energy) by using the α­(ε) relationship in eq 5. Doing this had effectively centered the LCL fit of all these systems to the same range, because the quoted *c*_1_ and *c*_2_ correspond to LCL results at a common *T* = 425 K. 425 K is a typical temperature where a majority of polymers would be found in their melt form, but even for polymers with *T*_g_ > 425 K, the application of eq 5 (with *c*_1_ and *c*_2_ for 425 K) is still justified because it corresponds to the “theoretical” equilibrium melt extrapolated below *T*_g_; see the Supporting Information of ref 87.^87^

In Table 1, we provide examples of LCL model characterizations covering a large set of polymers that were carried out by fitting to PVT data sets. Shown are the *r*, *v*, ε parameters and the average temperature (<*T*>) of the experimental data (in the melt state) that was used in that model fit. The pressure range of the data was typically 0 MPa up to about 100 MPa. Note that in the limit of low molecular weight (e.g., *M*_w_ ≤ 5000 g/mol), the oligomeric variants of these polymers will have experimental thermal expansivity values that become molecular weight dependent (with the experimental α increasing as *M*_w_ decreases). Correspondingly, LCL analysis of thermodynamic data in this low *M*_w_ limit will yield the expected difference in ε values, for example the strength of the segmental interactions will correspondingly weaken with decreasing *M*_w_. Also provided in Table 1 are the PVT data references, and the polymer full name/description and the corresponding acronyms. These acronyms cover most of those in the figures above; acronyms for several others (those that were not in the PVT data set fits in the table) can be found in ref 87. Finally, the polymers appearing in Figure 5b, from left to right, are as follows: PCLA, PBu, PEO, PVAc, PIB, PS, PLLA, PMMA. In Figure 7 the polymer and small molecule systems, in order of smallest *T*_g_ to largest, are PIB, PCLA, PEO, PVDF, PBu, PVAc, PPMA, PTBuA, PLLA, PBzMA, TPD, PEMA, P2VP, PS, P4MS, PMMA, PTBMA, P4ClS, P2ClS, TCTA, PTBS, TEL, PC, PVK; additional info on these systems can be found in refs 81 and 104.^104^

## References

[1] Flory, P. J. Principles of Polymer Chemistry; Cornell University Press: Ithaca, NY, 1953.

[2] Prigogine I., Trappeniers N., Mathot V. (1953). Statistical Thermodynamics
of R-Mers
and R-Mer Solutions. Discuss. Faraday Soc..

[3] Flory P. J., Orwoll R. A., Vrij A. (1964). Statistical
Thermodynamics of Chain
Molecule Liquids.I. Equation of State for Normal Paraffin Hydrocarbons. J. Am. Chem. Soc..

[4] Adam G., Gibbs J. H. (1965). On Temperature Dependence of Cooperative Relaxation
Properties in Glass-Forming Liquids. J. Chem.
Phys..

[5] Simha R., Somcynsky T. (1969). On Statistical Thermodynamics of Spherical and Chain
Molecule Fluids. Macromolecules.

[6] Sanchez I. C., Lacombe R. H. (1976). Elementary Molecular
Theory of Classical Fluids - Pure
Fluids. J. Phys. Chem. A.

[7] Dee G. T., Walsh D. J. (1988). A Modified Cell
Model Equation of State for Polymer
Liquids. Macromolecules.

[8] Chapman W. G., Gubbins K. E., Jackson G., Radosz M. (1989). Saft - Equation-Of-State
Solution Model for Associating Fluids. Fluid
Phase Equilib..

[9] Dudowicz J., Freed K. F., Madden W. G. (1990). Role of Molecular-Structure
on the
Thermodynamic Properties of Melts, Blends, and Concentrated Polymer-Solutions
- Comparison of Monte-Carlo Simulations with the Cluster Theory for
the Lattice Model. Macromolecules.

[10] Rodgers P. A. (1993). Pressure
Volume Temperature Relationships for Polymeric Liquids - a Review
of Equations of State and Their Characteristic Parameters for 56 Polymers. J. Appl. Polym. Sci..

[11] Kob W., Andersen H. C. (1995). Testing Mode-Coupling
Theory for a Supercooled Binary
Lennard-Jones Mixture - the Van Hove Correlation-Function. Phys. Rev. E.

[12] Sastry S., Debenedetti P., Stillinger F. (1998). Signatures of Distinct Dynamical
Regimes in the Energy Landscape of a Glass-Forming Liquid. Nature.

[13] Götze W. (1999). Recent Tests
of the Mode-Coupling Theory for Glassy Dynamics. J. Phys.-Condens. Matter.

[14] Long D., Lequeux F. (2001). Heterogeneous Dynamics
at the Glass Transition in van
Der Waals Liquids, in the Bulk and in Thin Films. Eur. Phys. J. E.

[15] Merabia S., Long D. (2008). Heterogeneous Dynamics and Pressure Dependence of the Dynamics in
van Der Waals Liquids. Macromolecules.

[16] Dudowicz J., Freed K. F., Douglas J. F. (2008). Generalized
Entropy Theory of Polymer
Glass Formation. Adv. Chem. Phys..

[17] Gnan N., Schroder T. B., Pedersen U. R., Bailey N. P., Dyre J. C. (2009). Pressure-Energy
Correlations in Liquids. IV. “Isomorphs” in Liquid Phase
Diagrams. J. Chem. Phys..

[18] Roland C. M. (2010). Relaxation
Phenomena in Vitrifying Polymers and Molecular Liquids. Macromolecules.

[19] Simmons D. S., Cicerone M. T., Zhong Q., Tyagi M., Douglas J. F. (2012). Generalized
Localization Model of Relaxation in Glass-Forming Liquids. Soft Matter.

[20] Starr F. W., Douglas J. F., Sastry S. (2013). The Relationship
of Dynamical Heterogeneity
to the Adam-Gibbs and Random First-Order Transition Theories of Glass
Formation. J. Chem. Phys..

[21] Dyre J. C. (2014). Hidden
Scale Invariance in Condensed Matter. J. Phys.
Chem. B.

[22] Cangialosi D. (2014). Dynamics and
Thermodynamics of Polymer Glasses. J. Phys.-Condens.
Matter.

[23] Xu W.-S., Freed K. F. (2014). Influence
of Cohesive Energy and Chain Stiffness on
Polymer Glass Formation. Macromolecules.

[24] Mirigian S., Schweizer K. S. (2015). Dynamical
Theory of Segmental Relaxation and Emergent
Elasticity in Supercooled Polymer Melts. Macromolecules.

[25] White R. P., Lipson J. E. G. (2016). Polymer Free
Volume and Its Connection to the Glass
Transition. Macromolecules.

[26] White R. P., Lipson J. E. G. (2017). Explaining the
T,V-Dependent Dynamics of Glass Forming
Liquids: The Cooperative Free Volume Model Tested against New Simulation
Results. J. Chem. Phys..

[27] Napolitano S., Glynos E., Tito N. B. (2017). Glass Transition
of Polymers in Bulk,
Confined Geometries, and near Interfaces. Rep.
Prog. Phys..

[28] Phan A. D., Schweizer K. S. (2018). Elastically Collective Nonlinear Langevin Equation
Theory of Glass-Forming Liquids: Transient Localization, Thermodynamic
Mapping, and Cooperativity. J. Phys. Chem. B.

[29] Grzybowski, A. ; Paluch, M. Universality of Density Scaling. In The Scaling of Relaxation Processes; Kremer, F. ; Loidl, A. , Eds.; Springer International Publishing: Cham, 2018; pp 77–119 10.1007/978-3-319-72706-6_4.

[30] Caruthers J. M., Medvedev G. A. (2018). Quantitative Model
of Super-Arrhenian Behavior in Glass
Forming Materials. Phys. Rev. Mater..

[31] White R. P., Lipson J. E. G. (2019). The Cooperative
Free Volume Rate Model for Segmental
Dynamics: Application to Glass-Forming Liquids and Connections with
the Density Scaling Approach. Eur. Phys. J.
E.

[32] Ginzburg V. V. (2021). Combined
Description of Polymer PVT and Relaxation Data Using a Dynamic “SL-TS2”
Mean-Field Lattice Model. Soft Matter.

[33] Ginzburg V.
V., Zaccone A., Casalini R. (2022). Combined Description of Pressure-Volume-Temperature
and Dielectric Relaxation of Several Polymeric and Low-Molecular-Weight
Organic Glass-Formers Using SL-TS2 Approach. Soft Matter.

[34] Lunkenheimer P., Loidl A., Riechers B., Zaccone A., Samwer K. (2023). Thermal Expansion
and the Glass Transition. Nat. Phys..

[35] Ginzburg V. V., Gendelman O. V., Zaccone A. (2024). Unifying Physical Framework for Stretched-Exponential,
Compressed-Exponential, and Logarithmic Relaxation Phenomena in Glassy
Polymers. Macromolecules.

[36] Roland C. M., Hensel-Bielowka S., Paluch M., Casalini R. (2005). Supercooled Dynamics
of Glass-Forming Liquids and Polymers under Hydrostatic Pressure. Rep. Prog. Phys..

[37] Schwartz G. A., Colmenero J., Alegria A. (2006). Pressure-Temperature Dependence of
Polymer Segmental Dynamics. Comparison between the Adam-Gibbs Approach
and Density Scalings. Macromolecules.

[38] Coslovich D., Roland C. M. (2009). Pressure-Energy
Correlations and Thermodynamic Scaling
in Viscous Lennard-Jones Liquids. J. Chem. Phys..

[39] Floudas, G. ; Paluch, M. ; Grzybowski, A. ; Ngai, K. Molecular Dynamics of Glass-Forming Systems - Effects of Pressure; Springer: Berlin, 2011.

[40] Fragiadakis D., Roland C. M. (2011). On the Density Scaling of Liquid
Dynamics. J. Chem. Phys..

[41] Bøhling L., Ingebrigtsen T. S., Grzybowski A., Paluch M., Dyre J. C., Schroder T. B. (2012). Scaling
of Viscous Dynamics in Simple Liquids: Theory,
Simulation and Experiment. New J. Phys..

[42] Mckenna G. B., Simon S. L. (2017). Challenges in the
Dynamics and Kinetics of Glass-Forming
Polymers. Macromolecules.

[43] Keddie J. L., Jones R. A. L., Cory R. A. (1994). Size-Dependent Depression
of the
Glass-Transition Temperature in Polymer-Films. Europhys. Lett..

[44] Ediger M. D., Forrest J. A. (2014). Dynamics near Free Surfaces and the
Glass Transition
in Thin Polymer Films: A View to the Future. Macromolecules.

[45] Richert R. (2011). Dynamics of
Nanoconfined Supercooled Liquids. Annu. Rev.
Phys. Chem..

[46] Alcoutlabi M., McKenna G. (2005). Effects of Confinement
on Material Behaviour at the
Nanometre Size Scale. J. Phys.-Condens. Matter.

[47] Adrjanowicz K., Kaminski K., Koperwas K., Paluch M. (2015). Negative Pressure Vitrification
of the Isochorically Confined Liquid in Nanopores. Phys. Rev. Lett..

[48] Simmons D. S. (2016). An Emerging
Unified View of Dynamic Interphases in Polymers. Macromol. Chem. Phys..

[49] Diaz-Vela D., Hung J.-H., Simmons D. S. (2018). Temperature-Independent
Rescaling
of the Local Activation Barrier Drives Free Surface Nanoconfinement
Effects on Segmental-Scale Translational Dynamics near Tg. ACS Macro Lett..

[50] White R. P., Lipson J. E. G. (2020). To Understand Film Dynamics Look
to the Bulk. Phys. Rev. Lett..

[51] Baschnagel J., Varnik F. (2005). Computer Simulations
of Supercooled Polymer Melts in
the Bulk and In-Confined Geometry. J. Phys.-Condens.
Matter.

[52] Cangialosi D., Alegria A., Colmenero J. (2016). Effect of
Nanostructure on the Thermal
Glass Transition and Physical Aging in Polymer Materials. Prog. Polym. Sci..

[53] Boucher V. M., Cangialosi D., Yin H., Schoenhals A., Alegria A., Colmenero J. (2012). T-g Depression
and Invariant Segmental
Dynamics in Polystyrene Thin Films. Soft Matter.

[54] Fukao K., Miyamoto Y. (2000). Glass Transitions and
Dynamics in Thin Polymer Films:
Dielectric Relaxation of Thin Films of Polystyrene. Phys. Rev. E.

[55] Priestley R. D., Cangialosi D., Napolitano S. (2015). On the Equivalence between the Thermodynamic
and Dynamic Measurements of the Glass Transition in Confined Polymers. J. Non-Cryst. Solids.

[56] Kremer F., Tress M., Mapesa E. U. (2015). Glassy
Dynamics and Glass Transition
in Nanometric Layers and Films: A Silver Lining on the Horizon. J. Non-Cryst. Solids.

[57] Kumar S. K., Benicewicz B. C., Vaia R. A., Winey K. I. (2017). 50th Anniversary
Perspective: Are Polymer Nanocomposites Practical for Applications?. Macromolecules.

[58] Cheng S., Carroll B., Bocharova V., Carrillo J.-M. Y., Sumpter B. G., Sokolov A. P. (2017). Focus: Structure
and Dynamics of the Interfacial Layer
in Polymer Nanocomposites with Attractive Interactions. J. Chem. Phys..

[59] Cheng S., Holt A. P., Wang H., Fan F., Bocharova V., Martin H., Etampawala T., White B. T., Saito T., Kang N.-G., Dadmun M. D., Mays J. W., Sokolov A. P. (2016). Unexpected
Molecular Weight Effect in Polymer Nanocomposites. Phys. Rev. Lett..

[60] Zhang W., Emamy H., Betancourt B. A. P., Vargas-Lara F., Starr F. W., Douglas J. F. (2019). The Interfacial
Zone in Thin Polymer
Films and around Nanoparticles in Polymer Nanocomposites. J. Chem. Phys..

[61] White R. P., Lipson J. E. G. (2023). Why Volume and Dynamics Decouple in Nanocomposite Matrices:
Space That Cannot Be Accessed Is Not Free. Phys.
Rev. Lett..

[62] Ferry, J. D. Viscoelastic Properties of Polymers, 2nd ed.; Wiley: New York, 1970.

[63] Williams M. L., Landel R. F., Ferry J. D. (1955). Mechanical
Properties of Substances
of High Molecular Weight.19. the Temperature Dependence of Relaxation
Mechanisms in Amorphous Polymers and Other Glass-Forming Liquids. J. Am. Chem. Soc..

[64] Plazek D. J. (1991). A Myopic
Review of the Viscoelastic Behavior of Polymers. J. Non-Cryst. Solids.

[65] Ricarte R. G., Shanbhag S. (2024). A Tutorial Review of Linear Rheology
for Polymer Chemists:
Basics and Best Practices for Covalent Adaptable Networks. Polym. Chem..

[66] Wang J.-s., Porter R. S. (1995). On the Viscosity-Temperature Behavior of Polymer Melts. Rheol. Acta.

[67] Cangialosi D., Boucher V. M., Alegria A., Colmenero J. (2013). Physical Aging
in Polymers and Polymer Nanocomposites: Recent Results and Open Questions. Soft Matter.

[68] Hutchinson J. M. (1995). Physical
Aging of Polymers. Prog. Polym. Sci..

[69] Hodge I. M. (1995). Physical
Aging in Polymer Glasses. Science.

[70] Kovacs, A. J. Transition vitreuse dans les polymères amorphes. Etude phénoménologique. In Fortschritte Der Hochpolymeren-Forschung; Springer: Berlin, Heidelberg, 1964; pp 394–507 10.1007/BFb0050366.

[71] Tool A. Q. (1946). Relation
Between Inelastic Deformability and Thermal Expansion of Glass in
Its Annealing Range. J. Am. Ceram. Soc..

[72] Narayanaswamy O. S. (1971). A Model
of Structural Relaxation in Glass. J. Am. Ceram.
Soc..

[73] Moynihan C. T., Macedo P. B., Montrose C. J., Gupta P. K., Debolt M. A., Dill J. F., Dom B. E., Drake P. W., Easteal A. J., Elterman P. B., Moeller R. P., Sasabe H., Wilder J. A. (1976). Structural
Relaxation in Vitreous Materials. Ann. N.Y.
Acad. Sci..

[74] Kovacs A., Aklonis J., Hutchinson J., Ramos A. (1979). Isobaric Volume and
Enthalpy Recovery of Glasses.2. Transparent Multi-Parameter Theory. J. Polym. Sci., Part B:Polym. Phys..

[75] Riechers B., Roed L. A., Mehri S., Ingebrigtsen T. S., Hecksher T., Dyre J. C., Niss K. (2022). Predicting
Nonlinear
Physical Aging of Glasses from Equilibrium Relaxation via the Material
Time. Sci. Adv..

[76] Di
Lisio V., Stavropoulou V.-M., Cangialosi D. (2023). Physical Aging
in Molecular Glasses beyond the Alpha Relaxation. J. Chem. Phys..

[77] Caporaletti F., Villanueva M. E., Molitor S., Zuo B., Napolitano S. (2026). Predicting
How Fast Crystals Grow at the Free Surface of Molecular Glasses. Mater. Horiz..

[78] Yang Y., Tian H., Napolitano S., Zuo B. (2023). Crystallization in
Thin Films of Polymer Glasses: The Role of Free Surfaces, Solid Interfaces
and Their Competition. Prog. Polym. Sci..

[79] Frantz P., Granick S. (1991). Kinetics of Polymer
Adsorption and Desorption. Phys. Rev. Lett..

[80] Napolitano S. (2020). Irreversible
Adsorption of Polymer Melts and Nanoconfinement Effects. Soft Matter.

[81] Song Z., Rodríguez-Tinoco C., Mathew A., Napolitano S. (2022). Fast Equilibration
Mechanisms in Disordered Materials Mediated by Slow Liquid Dynamics. Sci. Adv..

[82] Thoms E., Song Z., Wang K., Napolitano S. (2024). Simple Model
to Predict the Adsorption Rate of Polymer Melts. Phys. Rev. Lett..

[83] Li C., Napolitano S. (2025). Model Polymer
Systems Replicate the Experimental Features
of the Slow Arrhenius Process. Macromolecules.

[84] de
Gennes P. G. (1980). Dynamics of Fluctuations and Spinodal Decomposition
in Polymer Blends. J. Chem. Phys..

[85] Binder K. (1983). Collective
Diffusion, Nucleation, and Spinodal Decomposition in Polymer Mixtures. J. Chem. Phys..

[86] Higgins J. S., Cabral J. T. (2020). A Thorny Problem?
Spinodal Decomposition in Polymer
Blends. Macromolecules.

[87] White R. P., Napolitano S., Lipson J. E. G. (2025). Mechanistic Picture for the Slow
Arrhenius Process in Glass Forming Systems: The Collective Small Displacements
Model. Phys. Rev. Lett..

[88] White R. P., Lipson J. E. G. (2021). A Simple New
Way To Account for Free Volume in Glassy
Dynamics: Model-Free Estimation of the Close-Packed Volume from PVT
Data. J. Phys. Chem. B.

[89] Wolf S. E., Liu T., Govind S., Zhao H., Huang G., Zhang A., Wu Y., Chin J., Cheng K., Salami-Ranjbaran E., Gao F., Gao G., Jin Y., Pu Y., Toledo T. G., Ablajan K., Walsh P. J., Fakhraai Z. (2021). Design of a Homologous
Series of Molecular Glassformers. J. Chem. Phys..

[90] White R. P., Buculei D., Beale A. M. J. M., Goovaerts I., Keddie J. L., Lipson J. E. G. (2022). Spectroscopic
Ellipsometry as a Route
to Thermodynamic Characterization. Soft Matter.

[91] Beaucage G., Composto R., Stein R. (1993). Ellipsometric
Study of the Glass-Transition
and Thermal-Expansion Coefficients of Thin Polymer-Films. J. Polym. Sci., Part B:Polym. Phys..

[92] DeFelice J., Lipson J. E. G. (2014). Polymer Miscibility
in Supercritical Carbon Dioxide:
Free Volume as a Driving Force. Macromolecules.

[93] White R. P., Lipson J. E. G. (2015). Free Volume in
the Melt and How It Correlates with
Experimental Glass Transition Temperatures: Results for a Large Set
of Polymers. Acs Macro Lett..

[94] White R. P., Lipson J. E. G. (2025). How Local Structural
Change Affects Polymer Segmental
Dynamics: Crosslinks and Nanoparticles. Macromolecules.

[95] Schonhals, A. ; Kremer, F. Broadband Dielectric Spectroscopy; Kremer, F. ; Schonhals, A. , Eds.; Springer: Berlin, 2003.

[96] Floudas, G. Dielectric Spectroscopy. In Polymer Science: A Comprehensive Reference; Elsevier, 2012; pp 825–845 10.1016/B978-0-444-53349-4.00057-1.

[97] Debot A., White R. P., Lipson J. E. G., Napolitano S. (2019). Experimental
Test of the Cooperative Free Volume Rate Model under 1D Confinement:
The Interplay of Free Volume, Temperature, and Polymer Film Thickness
in Driving Segmental Mobility. Acs Macro Lett..

[98] Casalini R., Fragiadakis D., Roland C. M. (2011). Relaxation Dynamics of Poly­(Methyl
Acrylate) at Elevated Pressure. Macromolecules.

[99] Theobald S., Pechhold W., Stoll B. (2001). The Pressure
and Temperature Dependence
of the Relaxation Processes in Poly­(Methylmethacrylate). Polymer.

[100] Zoller, P. ; Walsh, D. Standard Pressure-Vol.-Temperature Data for Polymers; Technomic Pub Co.: Lancaster, PA, 1995.

[101] Casalini R., Roland C. M., Capaccioli S. (2007). Effect of
Chain Length on Fragility and Thermodynamic Scaling of the Local Segmental
Dynamics in Poly­(Methylmethacrylate). J. Chem.
Phys..

[102] Thoms E., Li C., Napolitano S. (2024). Tracing the
Slow Arrhenius Process Deep in the Glassy State–Quantitative
Evaluation of the Dielectric Relaxation of Bulk Samples and Thin Polymer
Films in the Temperature Domain. J. Chem. Phys..

[103] Thoms E., Napolitano S. (2023). Enthalpy-Entropy
Compensation in
the Slow Arrhenius Process. J. Chem. Phys..

[104] Caporaletti F., Napolitano S. (2024). The Slow Arrhenius
Process in Small
Organic Molecules. Phys. Chem. Chem. Phys..

[105] Wang B., Sanviti M., Alegria A., Napolitano S. (2023). Molecular
Mobility of Polymers at the Melting Transition. ACS Macro Lett..

[106] Teng Y., Guo Y. (2024). Multiple Relaxation Dynamics under
Electric Field Enables Tunable Viscoelastic Response of Poly­(Methyl
Methacrylate) above Glass Transition Temperature. J. Chem. Phys..

[107] Barzycki D. C., Ezzeddine D., Shanbhag S., Ricarte R. G. (2025). Linear
Viscoelasticity of Polystyrene Vitrimers: Segmental Motions and the
Slow Arrhenius Process. Macromolecules.

[108] Yuan H., Yan J., Gao P., Kumar S. K., Tsui O. K. C. (2022). Microscale Mobile
Surface Double Layer in a Glassy
Polymer. Sci. Adv..

[109] Yu H.-B., Wang Q. (2024). Liquid-like Clusters
in Glassy Solids
as a Unique State of Matter: Dissipative but Non-Diffusive. Mater..

[110] Wang K., Villanueva M. E., Caporaletti F., White R. P., Lipson J. E. G., Napolitano S., Losada-Pérez P. (2026). Glassy Dynamics as a Predictive Framework
for Lipid
Exchange Across Membranes. Small.

[111] Roy D., Roland C. M. (2013). Reentanglement Kinetics in Polyisobutylene. Macromolecules.

[112] Gu S.-Y., Zou C.-Y., Zhou K., Ren J. (2009). Structure-Rheology
Responses of Polylactide/Calcium Carbonate Composites. J. Appl. Polym. Sci..

[113] Masuda T., Kitagawa K., Onogi S. (1970). Viscoelastic
Properties
of Poly­(Methyl Methacrylates) Prepared by Anionic Polymerization. Polym. J..

[114] Luo P., Wen P., Bai H. Y., Ruta B., Wang W. H. (2017). Relaxation
Decoupling in Metallic Glasses at Low Temperatures. Phys. Rev. Lett..

[115] Krevelen†, D. W. van. ; Nijenhuis, K. te. Properties of Polymers: Their Correlation with Chemical Structure; Their Numerical Estimation and Prediction from Additive Group Contributions, 4th ed.; Elsevier Science: Amsterdam, 2009.

[116] White R. P., Lipson J. E. G., Higgins J. S. (2012). New Correlations
in Polymer Blend Miscibility. Macromolecules.

[117] White R.
P., Lipson J. E. G., Higgins J. S. (2012). How Pure Components
Control Polymer Blend Miscibility. Macromolecules.

[118] Wu R., Shaw M. T., Weiss R. A. (1992). Rheo-optical
Studies of Stress-induced
Phase Changes in Blends Exhibiting UCST: Polystyrene and Polyisobutylene. J. Rheol..

[119] Chen Z. J., Wu R.-J., Shaw M. T., Weiss R. A., Fernandez M. L., Higgins J. S. (1995). Rheo-Optical Behavior
of Binary Polymer
Blends: The Effect of Simple Shear Flow on Phase Behavior. Polym. Eng. Sci..

[120] White R. P., Lipson J. E. G. (2014). Free Volume,
Cohesive Energy Density,
and Internal Pressure as Predictors of Polymer Miscibility. Macromolecules.

[121] White R. P., Lipson J. E. G. (2018). Connecting Pressure-Dependent
Dynamics
to Dynamics under Confinement: The Cooperative Free Volume Model Applied
to Poly­(4-Chlorostyrene) Bulk and Thin Films. Macromolecules.

[122] White R. P., Lipson J. E. G. (2009). Chain Fluids:
Contrasts of Theoretical
and Simulation Approaches, and Comparison with Experimental Alkane
Properties. J. Chem. Phys..

[123] Roland C. M., McGrath K. J., Casalini R. (2006). Dynamic Heterogeneity
in Poly­(Vinyl Methyl Ether)/Poly­(2-Chlorostyrene) Blends. Macromolecules.

[124] Callaghan T. A., Paul D. R. (1993). Interaction Energies
for Blends of
Poly­(Methyl Methacrylate), Polystyrene, and Poly­(Alpha-Methylstyrene)
by the Critical Molecular-Weight Method. Macromolecules.

[125] Krishnamoorti R., Graessley W. W., Dee G. T., Walsh D. J., Fetters L. J., Lohse D. J. (1996). Pure Component Properties and Mixing
Behavior in Polyolefin Blends. Macromolecules.

[126] Yi Y., Zoller P. (1993). An Experimental and
Theoretical-Study of the Pvt Equation
of State of Butadiene and Isoprene Elastomers to 200-Degrees-C and
200-Mpa. J. Polym. Sci., Part B:Polym. Phys..

[127] Olabisi O., Simha R. (1975). Pressure-Volume-Temperature Studies
of Amorphous and Crystallizable Polymers.1. Experimental. Macromolecules.

[128] Kim C. K., Paul D. R. (1992). Interaction Parameters
for Blends
Containing Polycarbonates 1. Tetramethyl Bisphenol-a Polycarbonate
Polystyrene. Polymer.

[129] Ougizawa T., Dee G. T., Walsh D. J. (1991). Pressure
Volume
Temperature Properties and Equations of State in Polymer Blends -
Characteristic Parameters in Polystyrene Poly­(Vinyl Methyl-Ether)
Mixtures. Macromolecules.

